# Transcript analysis of the extended *hyp*-operon in the cyanobacteria *Nostoc *sp. strain PCC 7120 and *Nostoc punctiforme *ATCC 29133

**DOI:** 10.1186/1756-0500-4-186

**Published:** 2011-06-14

**Authors:** Marie Holmqvist, Pia Lindberg, Åsa Agervald, Karin Stensjö, Peter Lindblad

**Affiliations:** 1 Department of Photochemistry and Molecular Science, The Ångström Laboratories, Uppsala University, Box 523, SE-751 20 Uppsala, Sweden

## Abstract

**Background:**

Cyanobacteria harbor two [NiFe]-type hydrogenases consisting of a large and a small subunit, the Hup- and Hox-hydrogenase, respectively. Insertion of ligands and correct folding of nickel-iron hydrogenases require assistance of accessory maturation proteins (encoded by the *hyp*-genes). The intergenic region between the structural genes encoding the uptake hydrogenase (*hupSL*) and the accessory maturation proteins (*hyp *genes) in the cyanobacteria *Nostoc *PCC 7120 and *N. punctiforme *were analysed using molecular methods.

**Findings:**

The five ORFs, located in between the uptake hydrogenase structural genes and the *hyp*-genes, can form a transcript with the *hyp*-genes. An identical genomic localization of these ORFs are found in other filamentous, N_2_-fixing cyanobacterial strains. In *N. punctiforme *and *Nostoc *PCC 7120 the ORFs upstream of the *hyp*-genes showed similar transcript level profiles as *hupS *(hydrogenase structural gene), *nifD *(nitrogenase structural gene), *hypC *and *hypF *(accessory hydrogenase maturation genes) after nitrogen depletion. *In silico *analyzes showed that these ORFs in *N. punctiform*e harbor the same conserved regions as their homologues in *Nostoc *PCC 7120 and that they, like their homologues in *Nostoc *PCC 7120, can be transcribed together with the *hyp*-genes forming a larger extended *hyp-*operon. DNA binding studies showed interactions of the transcriptional regulators CalA and CalB to the promoter regions of the extended *hyp*-operon in *N. punctiforme *and *Nostoc *PCC 7120.

**Conclusions:**

The five ORFs upstream of the *hyp*-genes in several filamentous N_2_-fixing cyanobacteria have an identical genomic localization, in between the genes encoding the uptake hydrogenase and the maturation protein genes. In *N. punctiforme *and *Nostoc *PCC 7120 they are transcribed as one operon and may form transcripts together with the *hyp*-genes. The expression pattern of the five ORFs within the extended *hyp*-operon in both *Nostoc punctiforme *and *Nostoc *PCC 7120 is similar to the expression patterns of *hupS*, *nifD*, *hypF *and *hypC*. CalA, a known transcription factor, interacts with the promoter region between *hupSL *and the five ORFs in the extended *hyp*-operon in both *Nostoc *strains.

## Background

Cyanobacteria are an ancient group of organisms capable of both oxygenic photosynthesis and hydrogen evolution. Molecular hydrogen (H_2_) is produced by nitrogenases as a by-product when fixing atmospheric dinitrogen (N_2_) while uptake hydrogenases recapture the molecular hydrogen and oxidize it, to prevent energy losses from the cells. In addition, if present bidirectional hydrogenases have the capacity to both produce and oxidize H_2 _[1-3]. Hydrogenases and nitrogenases are oxygen sensitive enzymes [1,2] and therefore cyanobacteria have developed different strategies to maintain enzyme activity by separating the enzymes from oxygenic photosynthesis either spatially, e.g. in heterocysts [2,4,5], or temporally [5]. In heterocystous cyanobacteria nitrogenase and uptake hydrogenase are expressed in heterocysts, while the bidirectional hydrogenase is present in both heterocysts and vegetative cells. Neither the uptake nor the bidirectional hydrogenases are universally present among cyanobacteria. However, all filamentous strains examined so far capable of N_2_-fixation contain an uptake hydrogenase [2,3,6].

All cyanobacterial hydrogenases belong to the NiFe class of hydrogenases [3,6]. The uptake hydrogenase consists of a small and a large subunit, encoded by *hupS *and *hupL*, respectively [1,2,5]. The large subunit, HupL, harbors the Ni and Fe containing active site [3,6]. The assembly of the active site and correct folding of HupL is a complex process, requiring assistance of accessory proteins, encoded by at least *hypABCDEF*, and a hydrogenase specific protease, encoded by *hupW *[3,7]. The *hyp*-genes (*hyp *for *hy*drogenase *p*leiotropic) and the Hyp-proteins have been studied mainly in *Escherichia coli *[7]. However, mutational analyses in e.g. *Synechocystis *sp. PCC 6803 have shown that the corresponding cyanobacterial *hyp*-genes most likely have similar function [6,8].

The small subunit, HupS, mediates electron transport from the active site located in the large subunit to redox partners and downstream reactions through a set of FeS clusters [3,7]. Little is known about the cyanobacterial maturation process and assembly of FeS clusters in the small subunit. Three different kinds of FeS cluster biosynthesis systems have been identified in bacteria, the ISC (*i*ron-*s*ulphur *c*luster), SUF (mobilization of *su*l*f*ur) and NIF (*ni*trogen *f*ixation) system. They all have in common that they require a FeS cluster scaffolding protein (IscU and IscA, SufA, NifU) and a cysteine desulfurase (IscS, SufS, NifS), providing elemental sulphur [3,9]. Cyanobacteria lack homologues to *iscU *but the gene *nfu*, encoding a protein with high similarity to the C-terminal domain of NifU, is present. In cyanobacteria SufA and IscA seem to have a more regulatory function, e.g. sensing of redox stress and being involved in FeS cluster assembly under iron homeostasis. Nfu has been suggested to be involved in general FeS cluster assembly and is considered to be an essential FeS cluster scaffold protein [10].

There have been reports of proteins participating in the maturation process of the small subunit of uptake hydrogenases. The legume endosymbiont *Rhizobium leguminosarum *bv. viciae strain UPM791 harbors a subcluster of five genes, *hupGHIJK*, which has been connected to the maturation of the small subunit of the uptake hydrogenase. The subcluster is part of the hydrogenase gene cluster due to its location between the structural genes and the *hyp*-genes, and is preceded by a promoter (P_3_) upstream of *hupG *[11-13]. Homologues can be found in other aerobic, NiFe-type uptake hydrogenase containing bacteria such as *Azotobacter vinelandii, Rhodobacter capsulatus, Ralstonia eutropha *H16, *Azotobacter chroococcum *and *Bradyrhizobium japonicum *[14-16]. The presence of HupGHIJ appears to be connected to oxygen dependent microorganisms, since no homologues have been found in strictly anaerobic bacteria [11,16]. HupH forms a direct complex with the small subunit precursor, pre-HupS [11]. In addition, an interaction between the uptake hydrogenase small subunit Tat signal peptide and the HupH and HupG homologues, HoxQ and HoxO, has been shown in *Ralstonia eutropha *H16 [14,17,18]. The proposed functions of HupGHIJ in *R. leguminosarum *and HoxOQ in *R. eutropha *are to protect the FeS clusters in the small subunit from oxygen, stabilizing the small subunit complexes and preventing premature translocation of the small subunit to the periplasm by masking the Tat signal peptide [11,18].

**Figure 1:**
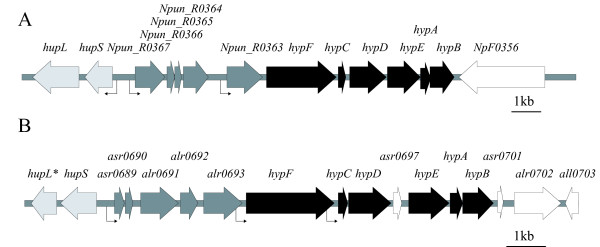
**Genomic arrangement of the ORFs upstream of the *hyp*-genes in *Nostoc punctiforme *ATCC 29133 and *Nostoc *sp. strain PCC 7120**. (A) In the filamentous, heterocyst forming cyanobacterial strain *N. punctiforme *the five ORFs upstream of the *hyp*-genes are located upstream of the uptake hydrogenase structural genes, *hupSL*, and in between *hupSL *and the *hyp*-genes, *hypFCDEAB*. (B) The same genomic arrangement can be found in the filamentous, heterocyst forming cyanobacterial strain *Nostoc *PCC 7120. This genomic arrangement of the ORFs upstream of the *hyp*-genes seems to be conserved in filamentous cyanobacteria harboring an uptake hydrogenase [19] * indicates the 5' end of *hupL *(encoding the N-terminal end of HupL) as it is annotated in vegetative cells. The identified tsps upstream of *hupSL *[36], *Npun_R0363 *[27] and *Npun_R0367 *(this work) in ATCC 29133 and upstream of *asr0689*, *hypF *and *hypC *in *Nostoc *PCC 7120 [19] are indicated by arrows.

Recently, we presented a set of putative additional *hyp*-genes in the cyanobacterium *Nostoc *sp. strain PCC 7120 (also called *Anabaena *sp. strain PCC 7120) [19]. These five ORFs, *asr0689 *to *alr0693*, are located in between *hupSL *and the *hyp*-genes (Figure 1, Table 1). The same genomic organization can be found in other N_2_-fixing strains such as the filamentous, heterocyst forming strains *Nostoc punctiforme *ATCC 29133, *Nodularia spumigena *CCY9414 and *Anabaena variabilis *ATCC 29413 as well as in the filamentous, non-heterocyst forming strains *Lyngbya majuscula *CCAP 1446/4 [19]. This genomic localization is identical to the organization of *hupGHIJK *discussed above. The conservation of these ORFs located upstream of the *hyp*-genes in N_2_-fixing cyanobacteria suggest that they may serve an important role in hydrogen metabolism, putatively in the maturation of the small subunit of the uptake hydrogenase. In *Nostoc *PCC 7120 the ORFs upstream of the *hyp*-genes can form a transcript together with the *hyp*-genes and a putative transcription start point (tsp) was identified upstream of *asr0689 *[19]. The ORFs upstream of the *hyp*-genes have been annotated as encoding unknown proteins. However, the deduced amino acid sequences harbor conserved regions such as tetratricopeptide repeats (TPR), NHL repeats (NHL from *N*CL-1, *H*T2A and *L*in-41) and a NifU like domain [19]. TPR domains are known to mediate protein-protein interactions [20-22], and NHL repeats may also have a similar function [23].

**Table 1:** The ORFs upstream of the *hyp*-genes in *Nostoc *sp. strain PCC 7120 and their homologues in *Nostoc punctiforme *ATCC 29133

*Nostoc *sp. strain PCC 7120	*asr0689*	*asr0690*	*alr0691*	*alr0692*	*alr069*
*Nostoc punctiforme *ATCC 29133	*Npun_R0366*	*Npun_R0365*	*Npun_R0367*	*Npun_R0364*	*Npun_R0363*

*N. punctiforme *and *Nostoc *PCC 7120 are two filamentous, heterocyst forming cyanobacterial strains used in the present study. Both possess an uptake hydrogenase while *Nostoc *PCC 7120 in addition possesses a bidirectional enzyme [2,24,25]. This makes the two strains interesting to compare due to the presence of only one set of *hyp*-genes in both genomes [19,26,27]. In this study we have further investigated the ORFs upstream of the *hyp*-genes by examining their transcript level pattern after nitrogen depletion in *N. punctiforme *and *Nostoc *PCC 7120. The results were compared to the transcript level pattern for genes known to be involved in hydrogen metabolism, such as *hupS *(encoding the uptake hydrogenase small subunit), *nifD *(encoding a nitrogenase subunit) and *hypC *and *hypF *(encoding Hyp-proteins). We also tested if the ORFs upstream of the *hyp*-genes in *N*. *punctiforme *may be, like in *Nostoc *PCC 7120, transcribed together with the *hyp*-operon. The ORFs upstream of the *hyp*-genes in *N. punctiforme *were analyzed *in silico *and the region examined experimentally for transcription start points. In addition, different DNA affinity studies were used to analyze binding of transcriptional regulators to the promoter region of the extended *hyp*-operon in both *N. punctiforme *and *Nostoc *PCC 7120.

## Methods

### Strains and culture conditions

The filamentous heterocystous cyanobacterial strains *Nostoc punctiforme *ATCC 29133 and *Nostoc *sp. strain PCC 7120 (also named *Anabaena *sp. strain PCC 7120) were cultured in BG11_0 _medium [28], sparged with air and grown at 25°C, at a continuous irradiance of 40 μmol of photons m^-2 ^s^-1 ^[29]. For non N_2_-fixing conditions the BG11_0 _medium was supplemented with 5 mM NH_4_Cl and 5 mM MOPS (pH 7.8).

### *In silico *genome analyses of the ORFs upstream of the *hyp*-genes

Sequence homology search for conserved domains in the ORFs upstream of the *hyp*-genes of *N. punctiforme *was performed in Cyanobase (http://genome.kazusa.or.jp/cyanobase/), UniProt Knowledgebase (Swiss-Prot and TrEMBL at http://www.expasy.org/sprot/) and Pfam (http://pfam.sanger.ac.uk). The deduced protein sequences of HupGHIJ were blasted against the *N. punctiforme *and *Nostoc *PCC 7120 and protein database at Cyanobase (http://genome.kazusa.or.jp/cyanobase) and National Center for Biotechnology Information (NCBI) (http://www.ncbi.nlm.nih.gov/).

### Heterocyst preparation

Heterocysts from *Nostoc *PCC 7120 were isolated as previously described with a few modifications [30,31]. The incubation step with lysozyme was prolonged to 2 hours and the sonication step decreased to 3 intervals of 10 s. The pellet, containing heterocysts, were washed 4 times in extraction buffer and centrifugated at 4°C at, in order, 1000 × g, 1000 × g, 750 × g, 500 × g and 250 × g for 5 min respectively.

### Nucleic acid isolation and analysis

Genomic DNA and RNA were isolated from *N. punctiforme *and *Nostoc *PCC 7120 cultures as previously described [19,29]. The rRNA quality was analyzed with the Experion System (Bio-Rad Laboratories) according to the manufacturer's instructions. The concentration was determined by absorbance measurements using Cary Win UV (Varian). Prior to RT-reactions RNA was treated with DNaseI (Fermentas) according to the instructions of the manufacturer.

### Primer construction

All oligonucleotides used are listed in Tables 2 and 3. Primers were designed either by Primer3 program (http://frodo.wi.mit.edu/primer3/) or manually. The secondary structure was analyzed with the Primer design utility program EazyPrimer™ (http://www.cybergene.se/EazyPrimer.htm) and the primers blasted against their corresponding *N. punctiforme *or *Nostoc *PCC 7120 genome at Cyanobase (http://genome.kazusa.or.jp/cyanobase/), to check their specificity.

**Table 2:** Primer oligonucleotides designed and used to generate or amplify cDNA from cyanobacterial cell cultures after nitrogen depletion

Primers	Sequence 5'-3'	Product size
***N. punctiforme *primers**		

*hupS *forward	ATTATGGCTACAAGGTGGTG	215 bp

*hupS *reverse	CAACACTGCCTTCAAATACC	

*nifD *forward	CCCAATGTGAAGATGAACCT	201 bp

*nifD *reverse	GCTGATACTTGGCGATAACT	

*rnpb *forward	AAGCAATAGCAACCATACAGA	198 bp

*rnpb *reverse	AATTGATCTGGCGGTATCTT	

*23S *forward	GAAACAGCCCAGACCACC	197 bp

*23S *reverse	AGTGAGCTATTACGCACTC	

*hypC *forward	AATCCCCGGACAAATTATAG	128 bp

*hypC *reverse	ATGTTTCTGCTGCTTCTTGT	

*hypF *forward	TTAAAACAGAAATTGTCCCT	216 bp

*hypF *reverse	CAACATCGTGGTATTCCTTT	

*Npun_0363 *forward	TATCATCAAGGACGGCTATT	197 bp

*Npun_0363 *reverse	TCTCCCCAAAAAGCGATGTC	

*Npun_0364 *forward	AACGGTGTGTAGCAGTAGG	179 bp

*Npun_0364 *reverse	TGGTGGTTTTGGTGGTTTG	

*Npun_0365 *forward	GGTTATCTGCCAAGTTATTT	190 bp

*Npun_0365 *reverse	TTGAAGAGAAACGGCATAAT	

*Npun_0366 *forward	TTAACAACGCCAAATCTGAT	220 bp

*Npun_0366 *reverse	CTAAGAAGATGGCAGGAATT	

*Npun_0367 *forward	AGATAGCACAGGGTTTTCAA	219 bp

*Npun_0367 *reverse	AAATCGGTAATGCCTCTTCA	

***Nostoc *PCC 7120 primers**		

*hupS *forward	TTGTTCAGGCAACACCATGT	154 bp

*hupS *reverse	GCCTAAGATGCAATCCCAAA	

*23S *forward	GCTAAGCGATGTACCGAAGC	199 bp

*23S *reverse	TAACCCAGAGTGGACGAACC	

*hypC *forward	GGAATCCCCGGACAAATTAC	200 bp

*hypC *reverse	GTTTCGGCTGCTTCTTGTTC	

*hypF *forward	GTGTCCCGAATGTGAGGACT	239 bp

*hypF *reverse	GCGTTGCATCACAAGCTAAA	

*asr0389 *forward	ATTTCTGATCAATATGGTCA	202 bp

*asr0389 *reverse	CCCAGACCCAAAACAATAGC	

*asr0390 *forward	CGGCGAGGTTATTTTTGAAG	196 bp

*asr0390 *reverse	TCTTTGAGAAGAAATGGCATGA	

*alr0391 *forward	TTGGCGAGGATAACCGATAG	218 bp

*alr0391 *reverse	CTGGGGTGGTCAATCAAGTT	

*alr0392 *forward	CAAGCTGCTTTGGAAGAGGT	203 bp

*alr0392 *reverse	ACCGCAATCACCGTCACAAT	

*alr0393 *forward	GGGACAGAATGCTAAGGGTA	200 bp

*alr0393 *reverse	CCCGATTTGGTTCATTCTCC	

*alr0394 *forward	CTTTGTTACAGAAGGGTGAA	213 bp

*alr0394 *reverse	CAGCAGGACTAACTAATAAC	

**RT primers *N. punctiforme***		

gsp*hypF*	TCGGATTTTCTTGACGAAGG	-

gsp*Npun_R0363*	TTCGATTATTTCCAGAATCAGC	-

gsp*Npun_R0364*	ATCAGGCACGGTTAAGCATT	-

Primers to amplify cDNA, generated with random primers, from cultures and isolated heterocysts of *Nostoc punctiforme *ATCC 29133 and cultures of *Nostoc *sp. strain PCC 7120 are shown as well as gene specific primers for generating cDNA from cultures of *Nostoc punctiforme *ATCC. Sizes of the resulting PCR product are shown in base pairs (bp). cDNA with gene specific primers were generated through reverse transcriptase reactions (RT).

**Table 3:** Primer oligonucleotides designed and used to locate novel transcription start points in *Nostoc punctiforme *ATCC 29133 and to amplify DNA probes for DNA affinity assay and electrophoretic mobility shift assay (EMSA) from *N. punctiforme *and *Nostoc *sp. strain PCC 7120 genomic DNA

Primers	Sequence 5'-3'	Product size	Biotinylated
*5'primer1 0367*	GGGGGTAATCCTCCCAAGTA	-	no

*5'primer2 0367*	TCACCACACATCTCGTAGGC	-	no

*5'primer3 0367*	TGCTGGGGGTCATAACTCTG	-	no

*N. punctiforme *F-bio	TTACGCATCTCATCACGGGCCA		yes

*N. punctiforme *R	ACAATACAAAAACACCTAGCCC	751 bp	no

*N*. 7120 F-bio	TGGCTATTAGTTTGTATATTGTT		yes

*N*. 7120 R	TCGCTATTACGTTCCTCTCT	500 bp	no

EMSA *PhupS *F	TTCTAAAATTCTAGGGGGAAATTG		no

EMSA *PhupS *R	GGGCTAGGTGTTTTTGTATTGT	558 bp	no

EMSA *hupL *F	CGCCATTATGAGGAAGCTGT		no

EMSA *hupL *R	CGGTCTTCATCCAACCAATC	308bp/1350 bp	no

Sizes of the resulting PCR products are shown in base pairs (bp)

### Transcript analysis

Reverse transcription (RT) reactions were performed according to the manufacturer's instructions with either the iScript™ cDNA Synthesis Kit (Bio-Rad Laboratories) containing random primers, using 0.25 μg total RNA from *N. punctiforme *and 0.5 μg total RNA from *Nostoc *PCC 7120, or the RevertAid™ First Strand cDNA Synthesis Kit (Fermentas) and gene specific primers, using 0.5 μg total RNA from *N. punctiforme*. The cultures were grown under either N_2_-fixing or non N_2_-fixing conditions. PCR amplifications using cDNAs of the respective genes were performed using corresponding primers (Table 2). To ensure that the PCR reaction of the reference gene, *23S*, were not saturated a semi-quantitative analysis of *23S *PCR product from *N. punctiforme *and *Nostoc *PCC 7120 were done by removing a sample every 2^nd ^cycle from, cycle 8 to 26, and comparing the amount of transcript (data not shown). PCR were used to amplify the regions between *hupS *to *Npun_R0367 *(using primers *hupS *forward and *Npun_R0367 *reverse), *Npun_R0367 *to *Npun_R0366 *(using primers *Npun_R0367 *forward and *Npun_R0366 *reverse), *Npun_R0366 *to *Npun_R0365 *(using primers *Npun_R0366 *forward and *Npun_R0365 *reverse), *Npun_R0365 *to *Npun_R0364 *(using primers *Npun_R0365 *forward and *Npun_R0364 *reverse), *Npun_R0364 *to *Npun_R0363 *(using primers *Npun_R0364 *forward and *Npun_R0363 *reverse) and *Npun_R0363 *to *hypF *(using primers *Npun_R0363 *forward and *hypF *reverse). Negative controls for the RT-reaction were RT-PCR on DNaseI treated RNA without RT-enzyme. Negative controls for the PCR reactions included PCR amplification without cDNA added and positive controls were made with genomic DNA from *N. punctiforme *and *Nostoc *PCC 7120 using the corresponding forward and reverse primer.

### PCR and agarose gel electrophoresis

PCR amplifications were carried out using the enhanced thermostable *Taq *DNA Polymerase DreamTaq™ (Fermentas), according to manufacturer's protocol, in a UnoCycler Thermal Cycler (VWR) according to the guidelines provided by the suppliers. For the transcript level analysis an annealing temperature of 60°C and 0.8 μl or 0.4 μl cDNA were used (*N. punctiforme *and *Nostoc *PCC 7120 respectively) in 10 μl reactions. The amount of cycles varied from 15 to 30 cycles for the different samples to avoid saturation of the PCR product in the reactions. The products were visualized on 1% agarose gels containing thiazole-orange. The gels were run with 1x sodium boric acid buffer at 160 V for 15 minutes.

### Identification of transcription start points

Transcription start points were located with the 5'RACE System for Rapid Amplification of cDNA Ends, Version 2.0 (Invitrogen), according to the manufacturer's instructions using 1 μg total RNA and gene specific primers (Table 3). The resulting PCR products were gel purified using the NucleoSpin^® ^Extract II (Macherey-Nagel) according to protocol and cloned into the pCR^® ^2.1-TOPO^® ^vector (Invitrogen), following to the manufacturer's instructions, before being sequenced at Macrogen Inc. The sequences were subsequently analyzed with BioEdit Sequence Alignment Editor 7.0.5.3. Negative controls included RT-PCR on DNaseI treated RNA without RT-enzyme, dC-tailing without TdT enzyme and PCR amplification without cDNA. Positive controls were made with genomic DNA from *N. punctiforme *using the corresponding forward and reverse primer.

### Protein extraction, DNA affinity assays and mass spectrometry

Cells from *N. punctiforme *and *Nostoc *PCC 7120 cultures were harvested by centrifugation at 3300 × g for 10 minutes at 4°C and washed once in protein buffer (100 mM Tris-HCl pH 7.5, 1 mM EDTA, 2 mM DTT, 0.5% Triton X-100, 10% glycerol and 1 mM PMSF). Proteins were extracted using 0.2 g of 0.6-mm-diameter glass beads and a Precellys^® ^24 homogenizer (Bertin Technologies) at a speed of 5500 for 6 × 30 s keeping samples on ice between runs. After centrifugation at 10000 × g for 10 minutes at 4°C, the supernatants were transferred to new tubes and subjected to further separation by centrifugation at 30000 × g for 20 minutes at 4°C. The supernatant was once more transferred to new tubes and the protein concentration was determined using a Bio-Rad Protein Assay Dye Reagent (Bio-Rad Laboratories) according to manufacturer's protocol. For the DNA affinity assay a DNA fragment stretching from ATG (translation start point) for *hupS *to ATG for *Npun_R0367 *in *N. punctiforme *(from now on referred to as P*hupS*/*Npun_R0367*) and DNA fragment stretching ATG for *hupS *to ATG for *asr0689 *for *Nostoc *PCC 7120 (from now on referred to as P*hupS*/*asr0689*), were amplified by PCR using the proofreading Phusion High Fidelity polymerase (Finnzymes) and the corresponding oligonucleotides (Figure 1, Table 3). The biotin labeled DNA fragments were incubated with streptavidin coated magnetic beads (Dynabeads^® ^M-280, Dynal Biotech) and subsequently with protein extracts as previously described [32]. For each reaction 1.5 μg DNA, 500 μg beads and 1000 μg proteins were used. Finally the beads were resuspended in 1x SDS loading buffer containing 62.5 mM Tris-HCl pH 6.8, 2% SDS, 2.5% 2-mercaptoethanol, 7,5 mM Ditiotreitol (DTT), 10% glycerol and 5 × 10^-3 ^% Bromphenol blue, and heated at 95°C for 5 minutes. The samples were excised from a 15% denaturing polyacrylamide gel, SDS-PAGE, stained with Brilliant Blue-G Colloidal (Sigma). Bands containing protein was cut from the gel and washed with water. The proteins were reduced by DTE and alkylated with iodoacetamide. After washing the proteins were digested with trypsin (Promega modified trypsin) in 50 mM NH4HCO3 at 37°C over night. Peptides were extracted and the extract was subjected to MALDI-Tof analysis using a Bruker Ultraflex-Tof/Tof instrument. Proteins were identified by using the peptide maps for search in the NCBInr database using the Mascot search engine (http://MatrixScience.com). If needed for identification, MS/MS analysis was performed using the same MS instrument and search engine. The regions upstream of *16S (Npun_r084 *and *rrn16Sc *for *N. punctiforme *and *Nostoc *PCC 7120, respectively) were used as a negative control as well as beads incubated without DNA but with protein extract.

### Electrophoretic mobility shift assay

For electrophoretic mobility shift assays, a fragment of the *N. punctiforme hupS *immediate upstream region was amplified by PCR using the proofreading Phusion High Fidelity polymerase (Finnzymes). The primers used were P*hupS *F and P*hupS *R yielding the 558 bp DNA fragment P*hupS*. Two control fragments, C1 and C2, were generated using primers against a region within the *hupL *gene, *hupL *F and *hupL *R. C1 was amplified with wild type *N. punctiforme *ATCC 29133 DNA as template, resulting in a 308 bp fragment, and C2 was amplified using template DNA where the *hupL *gene has been interrupted by an antibiotic resistance cassette [33], resulting in a 1350 bp fragment. Histidine-tagged *Nostoc *PCC 7120 CalA was expressed in *Nostoc *PCC 7120 as described previously [34]. After expression, the protein was purified on a Superflow Ni-NTA column (Qiagen) according to the manufacturer's instructions. Binding of the protein to the DNA fragments was performed as described previously [35], using 100 ng of each DNA fragment per 20 μl reaction, and different amounts, from 0 to 45 ng, of purified His-tagged CalA. After incubation at room temperature for 1 h, the assay mixtures were separated on an 8% non-denaturing polyacrylamide gel. The gel was subsequently stained with thiazole orange to visualize DNA bands.

## Results

### *In silico *genome analyses of the ORFs upstream of the *hyp*-genes demonstrates a conserved arrangement

To investigate if the same, or similar, conserved domains as reported for the five ORFs upstream of the *hyp*-genes in *Nostoc *PCC 7120 [19], *asr0689 *to *alr0693*, can be identified in the ORFs upstream of the *hyp*-genes in *N. punctiforme*, *Npun_R0367 *to *Npun_R0363 *(Figure 1), computational analyzes was performed. Indeed, in the deduced amino acid sequences of *Npun_R0366 *and *Npun_03*65, being the counterparts to *asr0689 *and *asr0690 *in *Nostoc *PCC 7120, putative transmembrane regions can be identified. Furthermore, in accordance to the findings in *Nostoc *PCC 7120 the deduced amino acid sequence of the *alr0691 *homologue *Npun_R0367 *and the *alr0693 *homologue *Npun_0363 *harbor TPR and NHL repeats, respectively. The deduced amino acid sequence of *alr0692 *was described as being similar to the C-terminal of NifU. When analyzing the homologue of alr0692 in *N. punctiforme *we found that *Npun_R0364 *as well was annotated as being similar to the C-terminal of *NifU *(Table 1).

### The ORFs upstream of the *hyp*-genes show the same pattern of transcription as the *hyp *genes

**Figure 2:**
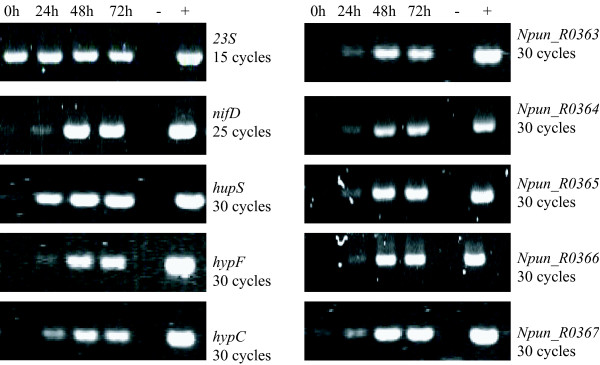
**Transcript levels of the ORFs upstream of the *hyp*-genes in *Nostoc punctiforme *ATCC 29133 after nitrogen depletion**. Agarose gels showing the amplified PCR products using cDNA prepared from RNA from *N. punctiforme *cultures 0, 24, 48 and 72 hours after nitrogen depletion The tested genes are the hydrogenase, nitrogenase and ribosome structural genes *hupS*, *nifD *and *23S*, the *hyp*-genes *hypC *and *hypF *and the ORFs upstream of the *hyp*-genes (*Npun_R0363*, *Npun_R0364*, *Npun_R0365*, *Npun_R0366 *and *Npun_R03673*). All DNA fragments were amplified with PCR using 30 cycles, except for *nifD *and *23S *where 25 and 15 cycles were used, respectively. Negative (-) and positive controls (+) for the PCR reactions are shown.

**Figure 3:**
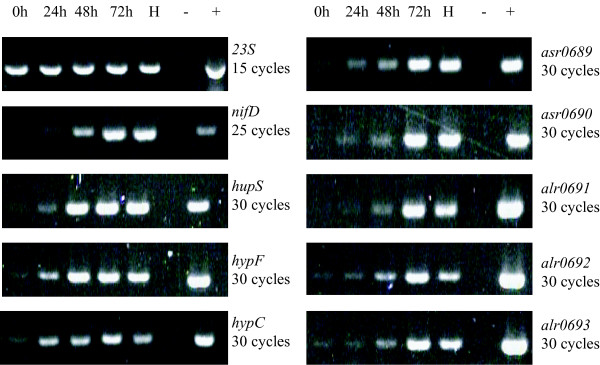
**Transcript levels of the ORFs upstream of the *hyp*-genes in *Nostoc *sp. strain PCC 7120 after nitrogen depletion**. Agarose gels showing the amplified PCR products using cDNA prepared RNA from *Nostoc *PCC 7120 cultures 0, 24, 48 and 72 hours after nitrogen depletion as well as isolated heterocysts 48 hours after nitrogen depletion. The tested genes are the hydrogenase and ribosome structural genes *hupS *and *23S*, the *hyp*-genes *hypC *and *hypF *and the ORFs upstream of the *hyp*-genes (*asr0689*, *asr0690*, *alr0691*, *alr0691 *and *alr0693*). All DNA fragments were amplified with PCR using 30 cycles, except for *nifD *and *23S *where 25 and 15 cycles were used, respectively. Negative (-) and positive controls (+) for the PCR reactions are shown.

To investigate if the ORFs upstream of the *hyp*-genes are expressed in the same manners as the *hyp*-genes and the structural genes of the hydrogenase and the nitrogenase, RNA was preparated from *N. punctiforme *and *Nostoc *PCC 7120 cultures 0, 24, 48 and 72 hours after nitrogen depletion, and from *Nostoc *PCC 7120 heterocysts 48 hours after nitrogen depletion. Note that the 0 hour sample represents a non N_2_-fixing condition. The control gene, *23S*, showed, as expected, a constitutive high transcript level in both strains while the transcript levels of the structural genes *nifD*, *hupS *and the *hyp*-genes *hypF *and *hypC *were upregulated 24 hours after transition to N_2_-fixing conditions (Figures 2, 3). The five ORFs located upstream of the *hyp*-genes in both organisms showed similar transcript level patterns as *nifD*, *hupS*, *hypF *and *hypC *after nitrogen depletion. The presence of transcript in RNA samples from heterocyst preparations showed that the expression takes place in the heterocyst and hence could be related to the expression of the uptake hydrogenase in *Nostoc *PCC 7120. In *N. punctiforme *the highest transcript levels for most genes occurred 48 hours after nitrogen depletion while the highest transcript levels for most genes were found 72 hours after nitrogen depletion in *Nostoc *PCC 7120. The expression of *23S*, used as a control representing a non-induced transcript, was constant for all time points in both strains. All the negative controls for the RT- reaction were blank (data not shown).

### The genes upstream of the *hyp*-genes can be transcribed together with the *hyp*-genes as one operon

**Figure 4:**
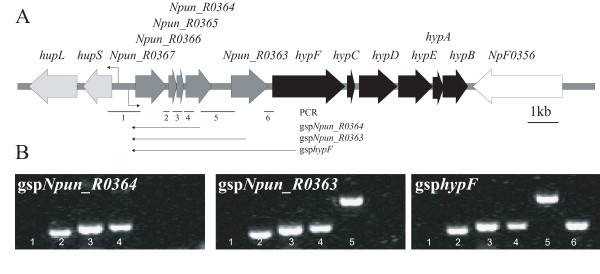
**The genes upstream of the *hyp*-genes can be transcribed together with the *hyp*-genes in *Nostoc punctiforme *ATCC 29133**. (A) Physical map of the *hyp*-genes and the ORFs upstream of the *hyp*-genes in *Nostoc punctiforme *ATCC 29133. The tsp upstream of *hupSL *[27] and the identified tsp upstream of *Npun_R0367 *are indicated by arrows. The regions amplified by RT-PCR, using the gene specific primers gsp*Npun_R0364*, gsp*Npun_R0363 *and gsp*hypF*, are depicted as arrows under the map and the regions amplified by PCR are depicted as numbered lines under the map. (B) PCR amplification of regions 1-4, 1-5 and 1-6 from cDNA generated with the gene specific primers gsp*Npun_R0364*, gsp*Npun_R0363 *and gsp*hypF*, respectively.

To investigate if the five ORFs upstream of the *hyp*-genes, like in *Nostoc *PCC 7120, can form a transcript together with the *hyp*-genes regions between the genes were amplified from cDNA from N_2_-fixing cultures of *N. punctiforme *using gene specific primers for the individual genes. The results show that the region between *hupS *and *Npun_R0367 *can not be amplified. However, when using the gene specific primer *Npun_R0364 *for cDNA synthesis the intergenic regions between *Npun_R0367*-*Npun_R0366*, *Npun_R0366*-*Npun_R0365*, and *Npun_R0365*-*Npun_R0364 *can be amplified. In addition, the region between *Npun_R0364*-*Npun_R0363 *and *Npun_R0363*-*hypF *can be amplified using the gene specific primers *Npun_R0363 *and *hypF *respectively for the cDNA synthesis (Figure 4). A tsp could be identified 170 bp upstream of *Npun_R0367*. In the promoter region a putative -10 box was found (TATAGT) and possibly an imperfect -35 box (TAGAAT). The previously reported NtcA binding site [36] is centered -64.5 bp upstream of the tsp.

### CalA is binding to the *hupSL Npun_R0367 *promoter region in *N. punctiforme *and the *hupSL asr6890 *promoter region in *Nostoc *PCC 7120

**Figure 5:**
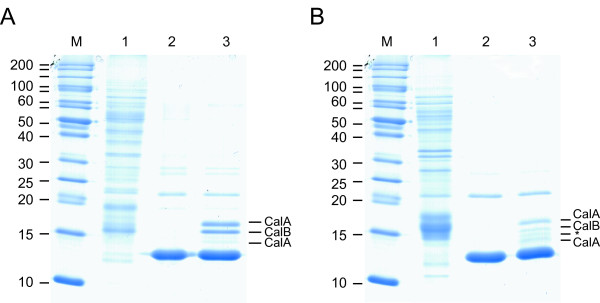
**DNA affinity assay of the *hupS*/*Npun_R0367 *promoter region from *Nostoc punctiforme *ATCC 29133 and the *hupS*/*asr0389 *promoter region from *Nostoc *sp. strain PCC 7120 and total protein extract from respective strain**. SDS-PAGE of proteins interacting with (A) the *hupS*/*Npun_R0367 *promoter region from *N. punctiforme *and (B) the *hupS*/*asr0389 *promoter region from *Nostoc *PCC 7120 from DNA-protein affinity assays. Lanes: M) protein molecular weight marker; 1) Total protein extract, 2) DNA-free negative control, 3) *hupS*/*Npun_R0367 *or *hupS*/*asr0389 *promoter region respectively. The unlabelled bands on the gel, present in both negative controls and samples, correspond to identified peptides either from unspecific binding, e.g. phycobilisome linker polypeptide (weak bands), artifacts from the experimental procedure, e.g. streptavidin (strongest band) or peptides with too low concentration to be identified (*).

**Figure 6:**
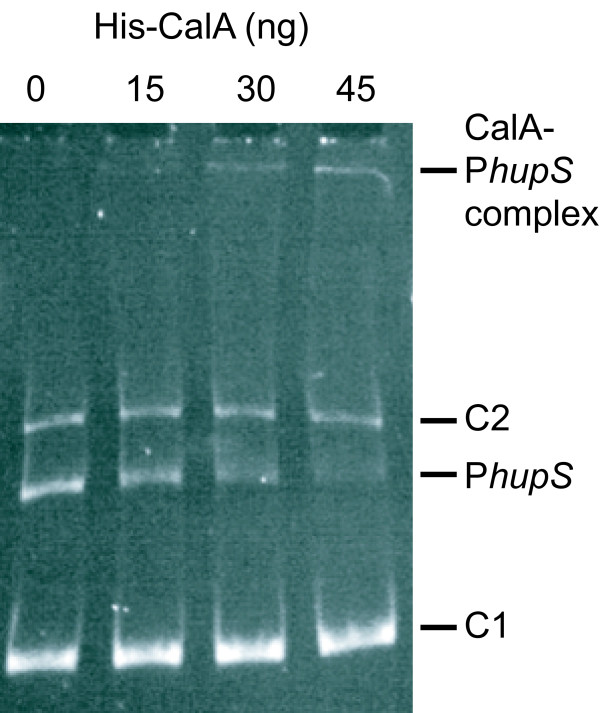
**Electrophoretic mobility shift assay of the hupS/*Npun_R0367 *promoter region in *Nostoc punctiforme *ATCC 29133**. Electrophoretic mobility shift assay showing specific binding of purified CalA to the *N. punctiforme hupS *upstream region. C1 - 308 bp control fragment, C2 - 1350 bp control fragment, P*hupS *- 558 bp *hupS *immediate upstream region fragment. 100 ng of each fragment and increasing amounts (see label for each lane) of purified histidine-tagged CalA (His-CalA) from *Nostoc *PCC 7120 were used in the reaction mixtures.

In order to identify potential transcriptional regulators interacting with the intergenic region harboring the *hupS*/*Npun_R0367 *promoter regions in *N. punctiforme *and the intergenic region harboring the *hupS *and the *asr6890 *promoter region in *Nostoc *PCC 7120, DNA-protein affinity assays were performed and proteins interacting with the promoter region fished out from a total protein extract of N_2_-fixing cultures. The resulting samples were run on a SDS-PAGE, bands were excised and the proteins analyzed with mass spectrometry. Among the identified peptides were CalA (encoded by *Npun_R5944*, *alr0946*) and CalB (encoded by *Npun_R2896*, *all2080*). The rest of the identified peptides, present in both negative controls and samples, were either from unspecific binding, e.g. phycobilisome linker polypeptide, or artifacts from the experimental procedure, e.g. streptavidin [see [32,37]]. The DNA affinity assay demonstrated an interaction of CalA and CalB to the intergenic region harboring the promoter region of the extended *hyp*-operon and the *hupSL *promoter region in *N. punctiforme *as well as in *Nostoc *PCC 7120 (Figure 5). However, using this technique it is not possible to rule out that only one of the proteins is binding to the DNA and the second protein is interacting with the DNA binding protein. The expected sizes for CalA and CalB are approximately 16.2 and 15.3 kDa in *N. punctiforme *and 16.1 and 15.3 in *Nostoc *PCC 7120, respectively. Those sizes matches the sizes read from the gel with the exception of the lowest CalA band for *Nostoc *PCC 7120. Neither CalA nor CalB showed any interaction with the negative controls (16S data not shown). In order to confirm specific binding of CalA to the *N. punctiforme hupS/Npun_R0367 *promoter region, electrophoretic mobility shift assays were performed. A fragment of *N. punctiforme *DNA stretching from -1 to -558 bp upstream of the *hupS *start codon, was amplified by PCR and mixed with purified, histidine-tagged CalA from *Nostoc *PCC 7120 [34]. The CalA proteins from *Nostoc *PCC 7120 and *N. punctiforme *differ in only four positions of their predicted amino acid sequences (data not shown), and therefore, we expect that the purified *Nostoc *PCC 7120 CalA could substitute for *N. punctiforme *CalA in these experiments. To investigate non-specific binding of the protein to DNA, two control fragments, one a 308 bp part of the *hupL *gene, and the other the same part of *hupL *interrupted by an antibiotic resistance cassette, resulting in a 1350 bp fragment, were also included. When the reaction mixtures were separated by non-denaturing PAGE, the 558 bp DNA fragment from the *hupS *upstream region was retarded on the gel (Figure 6), while the two control fragments were not, demonstrating specific binding of CalA to the *hupS *upstream fragment. Electrophoretic mobility shift assays performed with the complete *hupS/Npun_R0367 *promoter region, 751 bp, showed the same specific binding of CalA (data not shown).

## Discussion

Here we show that the five ORFs upstream of the *hyp*-genes in *N. punctiforme *harbors the same conserved regions as reported for *Nostoc *PCC 7120 [19]. The homologues *alr0691 *and *Npun_R0367 *contains TPR domains [19]. TPR domains mediate protein-protein interactions and are evolutionary conserved. They have been found in a variety of proteins involved in a wide range of cellular processes, for example protein folding. The numbers of TPR repeats differ between proteins, normally 3-16, and they are not connected to any specific position in primary protein sequences [20-22]. Likewise the homologues *alr0639 *and Npun_R0363 harbor NHL repeats also believed to be involved in protein-protein interactions [23]. FeS clusters are important for electron transfer to and from the active site in hydrogenases. Assembly and insertion of FeS clusters is a complex process; Fe and S atoms must be mobilized from storages, transported to the correct cellular localization and inserted into the correct apoprotein. In the three FeS cluster assembly systems found so far the assistance of a scaffold protein and a cysteine desulfurase is needed [3,9]. Interestingly, homologue genes *alr0692 *and *Npun_R0364 *contain a conserved domain that is similar to the C-terminal domain of NifU. Proteins of the NifU family function as a scaffolds for FeS cluster assembly but another protein family, Nfu, with sequence similarity to the C-terminal domain of NifU has also been reported to function as biosynthetic scaffolds [3]. In cyanobacteria, Nfu have been proposed to be the primary FeS cluster scaffold protein [10].

The transcript level analysis of the ORFs upstream of the *hyp*-genes showed, as expected, an upregulation of transcripts for the control genes, *nifD *and *hupS *(nitrogenase and uptake hydrogenase structural genes) and *hypF *and *hypC *(hydrogenase maturation genes) when going from non N_2 _to N_2_-fixing conditions in both *N. punctiforme *and *Nostoc *PCC 7120. This increase in transcript levels was expected and has been shown before [6,27]. Similarly, an upregulation of the transcript levels for all five ORFs upstream of the *hyp*-genes were seen after nitrogen depletion in both organisms while the amount of transcript of the control, *23S *(ribosomal subunit), were constant. For *Npun_0363 *this is in accordance with the earlier finding that this gene is expressed as one transcript with the *hyp*-genes [27]. *Nostoc *PCC 7120 harbors a bidirectional hydrogenase. To distinguish if an increase in transcript levels after a shift to N_2_-fixing conditions was connected to hydrogen metabolism in the heterocysts and not to bidirectional hydrogenase activity in the vegetative cells, heterocysts were isolated 48 hours after nitrogen depletion. The presence of transcripts of all genes tested in the heterocyst sample strongly indicates that expression takes place in the heterocysts. Since the ORFs upstream of the *hyp*-genes are not present in cyanobacterial strains containing only the bidirectional hydrogenase this points to a function related to the uptake hydrogenase. In *Nostoc *PCC 7120 the highest transcript levels of most genes investigated were detected 72 hours after nitrogen depletion while the highest transcript levels of most genes were detected already after 48 hours in *N. punctiforme*. The five ORFs upstream of the *hyp*-operon in *N. punctiforme *were shown to have, as *hupGHIJK *in *R. leguminosarum *[12], a tsp located upstream the gene cluster. In addition, as in *Nostoc *PCC 7120 [19], they are transcribed as a single operon and can form a transcript together with the *hyp*-genes in an extended *hyp*-operon. In bacterial genomes, genes that are functionally related are often clustered together and transcribed from the same promoter. By coordinating protein activities with gene regulation, genes expressed during different growth conditions, e.g. N_2_-fixation, can be turned on and off efficiently with minimal energy expenditure [38].

In *Nostoc *PCC 7120 a putative tsp was found upstream of *asr0689 *and in the promoter region a putative NtcA binding site and an extended -10 box were identified. In addition, tsps upstream of *hypF *and *hypC *were identified suggesting transcripts of varying sizes [19]. Occurrence of several tsps in one operon has been shown before in cyanobacteria and might allow for a more fine tuned regulation [39,40]. In *N. punctiforme *a tsp has been identified upstream of *Npun_R0363 *together with a putative -10 box and a putative NtcA binding site. No tsp has been found upstream of *hypF *or *hypC *[27]. In this study we found a novel tsp 170 bp upstream of the translation start point, ATG, of *Npun_R0367 *as well as a - 10 and possibly a -35 box. NtcA is a global regulator of nitrogen metabolism in cyanobacteria [41,42]. The -10 box in the *Npun_R0367 *promoter match the consensus sequence (TAN_3_T) reported for Class II NtcA activated promoters [41,42], but there is no NtcA binding site overlapping the area of the -35 box. There is, however, a NtcA binding site located further upstream and we have earlier reported that NtcA does indeed interact specifically in EMSAs with this proposed binding site [43]. This NtcA binding motif was first associated transcription of *hupSL *and is centered at -258.5 in the *hupSL *promoter [36]. However, the promoter regions of *hupSL *and *Npun_R0367 *are located in the same intergenic region, although in opposite direction (Figure 1). In the *Npun_R0367 *promoter region the NtcA binding site is centered at - 64.5 from the tsp. This shorter distance from the NtcA binding site to the tsp fits better, although not perfect, with the -41.5 distance reported for NtcA regulated promoters [41]. Interestingly, 10.5 base pairs approximately correspond to one helical turn of the DNA [23] which means that the NtcA site will have almost the reported angular orientation compared to the Npun_R0367 tsp. This, together with the importance of the NtcA binding site to the transcription of *hupSL *being low [43], supports our earlier suggestion that NtcA might not be involved in the regulation of *hupSL *but rather *Npun_R0367*, or putatively that NtcA is involved in the regulation of transcription from both promoters [43].

DNA binding assays showed that the novel transcriptional regulator CalA interacts specifically with the promoter region of the extended *hyp*-genes in both *N. punctiforme *and *Nostoc *PCC 7120 (Figure 5). Interestingly, two different bands were both identified as CalA. They may be either intact, cleaved, or partly degraded CalA, or different forms of CalA Furthermore, the CalA homologue, CalB, is also binding to the extended *hyp *promoter regions, or alternatively to CalA, in the respective strains (Figure 5). The cyanobacterial AbrB like Cal proteins belong to the transcription regulator family AbrB (Antibiotic resistance). They have a conserved DNA binding region in the C-terminal part of the protein, and the cyanobacterial Cal proteins may be classified into two separate clades, CalA and CalB [34,44]. AbrB is a global transition-state regulator well studied in *Bacillus subtilis *[44], that directly, or indirectly, regulates more than 60 different genes [45]. No consensus sequence for AbrB binding has been identified, instead the protein is believed to recognize a specific DNA 3D-conformation [46,47] and regulating by binding to promoter regions with different affinities [45] thereby fine-tuning the regulatory process. The functions of CalA and CalB in cyanobacteria are largely unknown, but parts of it are starting to be revealed. CalA appears to be essential, since no attempts to create a fully segregated knock-out mutant have been successful [37,44], and have been shown to interact with the promoter regions of genes involved in hydrogen metabolism in *Nostoc *PCC 7120 [34] and *Synechocystis *PCC 6803 [37], toxin production in *Aphanizomenon ovalisporum *[48], and with the promoter regions of FtsZ [49] and FeSOD in *Nostoc *PCC 7120 [50]. Fully segregated knock-out mutants of CalB have, however, been constructed in *Synechocystis *PCC 6803 [44] and it appears to play an important role in the regulation of genes involved in nitrogen uptake [44] and carbon concentrating mechanisms [51]. Since CalA interacts with the promoter region of *hypC *in *Nostoc *PCC 7120 [34] it makes sense that CalA also is involved in the regulation of the upstream of the *hyp*-operon in *N. punctiforme *and *Nostoc *PCC 7120. Furthermore, due to the fact that the genes in the extended *hyp*-operon are expressed during nitrogen depletion, together with many other genes involved in N_2_-fixation and nitrogen metabolism, interaction of CalB with the extended *hyp *promoter region is not unreasonable. Whether CalA or CalB activates or represses transcription of the extended *hyp*-operon in *N. punctiforme *and *Nostoc *PCC 7120 remains to be revealed.

## Conclusions

The five ORFs upstream of the *hyp*-genes in several filamentous N_2_-fixing cyanobacteria have an identical genomic localization, in between the uptake hydrogenase structural genes, *hupSL*, and the maturation protein genes, *hypABCDEF*. These ORFs are not present in strains harboring only the bidirectional hydrogenase. In *N. punctiforme *and *Nostoc *PCC 7120 they are transcribed as one operon and may form transcripts together with the *hyp*-genes. The homologues *alr0691 *and *Npun_R0364 *both contain a domain that is similar to NifU, a FeS cluster scaffold protein believed to be involved in the maturation of FeS cluster containing subunits. The expression pattern of the five ORFs within the extended *hyp*-operon in both *Nostoc punctiforme *and *Nostoc *PCC 7120 is similar to the expression patterns of *hupS*, *nifD*, *hypF *and *hypC*. CalA, a known transcription factor, interacts with the promoter region between *hupSL *and the five ORFs within the extended *hyp*-operon in both *Nostoc *strains.

## Author competing interests

The authors declare that they have no competing interests.

## Authors' contributions

MH performed most of the experimental work and was involved in designing the experiments, analyzing the data and writing the manuscript. PiL carried out the electrophoretic mobility shift assays and was involved in writing of the manuscript. ÅA conceived the project and was involved in designing the experiments, and was involved in the transcriptional analysis. KS supervised the experimental work, participated in its design, and was involved in writing of the manuscript. PL coordinated the project and the writing of the manuscript. All authors have read and approved the manuscript.
